# Costs and effects of a 'healthy living' approach to community development in two deprived communities: findings from a mixed methods study

**DOI:** 10.1186/1471-2458-11-25

**Published:** 2011-01-11

**Authors:** Helen A Snooks, Bridie Angela Evans, David Cohen, Michael Nugent, Frances Rapport, Jon Skone, Angie Meredith, Tricia Davies, Diana O'Sullivan

**Affiliations:** 1 CHIRAL, College of Medicine, Swansea University, Singleton Park, Swansea, SA2 8PP, UK; 2 Department of Health, Sport and Science, Glamorgan University, Pontypridd, CF37 1DL, UK; 3 Third Sector First, Bridleway House, Newchurch, Rossendale, Lancashire, BB4 9DR, UK; 4 Pembrokeshire County Council, County Hall, Haverfordwest, SA61 1TP, UK; 5 Community Researcher c/o Swansea University SA2 8PP, UK

## Abstract

**Background:**

Inequalities in health have proved resistant to 'top down' approaches. It is increasingly recognised that health promotion initiatives are unlikely to succeed without strong local involvement at all stages of the process and many programmes now use grass roots approaches. A healthy living approach to community development (HLA) was developed as an innovative response to local concerns about a lack of appropriate services in two deprived communities in Pembrokeshire, West Wales. We sought to assess feasibility, costs, benefits and working relationships of this HLA.

**Methods:**

The HLA intervention operated through existing community forums and focused on the whole community and its relationship with statutory and voluntary sectors. Local people were trained as community researchers and gathered views about local needs though resident interviews. Forums used interview results to write action plans, disseminated to commissioning organisations. The process was supported throughout through the project.

The evaluation used a multi-method before and after study design including process and outcome formative and summative evaluation; data gathered through documentary evidence, diaries and reflective accounts, semi-structured interviews, focus groups and costing proformas. Main outcome measures were processes and timelines of implementation of HLA; self reported impact on communities and participants; community-agency processes of liaison; costs.

**Results:**

Communities were able to produce and disseminate action plans based on locally-identified needs. The process was slower than anticipated: few community changes had occurred but expectations were high. Community participants gained skills and confidence. Cross-sector partnership working developed. The process had credibility within service provider organisations but mechanisms for refocusing commissioning were patchy. Intervention costs averaged £58,304 per community per annum.

**Conclusions:**

The intervention was feasible and inexpensive, with indications of potential impact at individual, community and policy planning levels. However, it is a long term process which requires sustained investment and must be embedded in planning and service delivery processes.

## Background

Inequalities in health have proved internationally resistant to 'top down' approaches. It is increasingly recognised that health promotion initiatives are unlikely to succeed without strong local involvement at all stages of the process [1-4] and many programmes across developed and developing societies now use grass roots approaches [5-10]. This paper reports results from an evaluation of a healthy living approach to community development (HLA), developed as an innovative response to local concerns about a lack of appropriate services, in two Pembrokeshire communities affected by economic decline, social exclusion and poor health.

The study was funded through the Welsh Assembly Government's Sustainable Health Action Research Programme (SHARP) which aimed to *"support and provide evidence on the effectiveness of interventions in health determinants" *[11]p31, focusing on communities with the highest incidence of ill health and social exclusion. Projects funded through this programme were based on collaborations between public, private, voluntary and academic sectors working with local communities with an aim of providing evidence of:

• effective/ineffective practice in addressing health determinants

• impact on health inequalities

• influence on social capital and/or social cohesion

• potential role of communities and partnerships in these issues [11]

Evaluation of community based initiatives is essential if policy and local practice are to be based on evidence about what works, and how. Evidence about effectiveness of community development approaches to addressing health related issues is limited [12]. However, traditional evaluation designs used to generate evidence of effectiveness for medical interventions such as drugs are generally unsuitable for application in the community context [13]. Setting up and carrying out a randomised controlled trial to evaluate a community based public health or health promotion intervention, whilst not impossible, is acknowledged internationally as likely to be expensive and potentially inappropriate [4,14]. In particular, effects are often overlapping with those of other diffuse initiatives in the same area. In addition, exact processes and outcomes are difficult to specify in advance, as the intervention itself is subject to change [15]. Innovative approaches to evaluation that are increasingly being used include an action research model of participatory implementation alongside evaluation [16-18].

To evaluate an explicitly community focused 'healthy living' intervention in West Wales, a participative and multi-method approach was therefore taken. The evaluation aimed to produce evidence concerning change and the costs of change that would inform developments elsewhere, through the generation of evidence with wider relevance, and through the building of theory. The action research approach adopted for the study aimed to bring about change as well as produce information about the nature, extent and costs of process and impact [12].

### The intervention

The Health Living Approach (HLA) was a community-led process of identifying and addressing issues affecting local health and wellbeing. The approach operated through existing community forums in areas where deprivation levels were associated with reduced levels of health and wellbeing [19-21]. Support was provided by the HLA project Action Researcher (AR) to enable partnership working between community, statutory and voluntary sectors through a Project Steering Group (PSG). Local people were recruited and trained as community researchers to carry out interviews with residents in their own communities to gather views about local needs and priorities related to health and wellbeing. The forums used the interview results to write plans recommending ways forward which were disseminated to statutory and voluntary organisations. The AR provided support throughout the process by encouraging and facilitating development of new skills and opportunities for community members, forums and through the multi-sector PSG.

Characteristics of the Healthy Living Approach are as follows:

• Local people are recruited and trained to work as community researchers

• Community researchers interview fellow residents about local issues affecting their health and wellbeing

• Interview results are reported back to communities who give feedback on results and ideas for action

• Community forums prepare action plans

• Action plan dissemination events are held with organisations and agencies responsible for issues raised

• **Throughout**: the action researcher supports community forums to coordinate and communicate across the statutory - voluntary - community partnership

### Ethical approval

Standards of research governance were adhered to throughout the study. Appropriate consent was obtained prior to data collection, records were stored securely and confidentiality of study participants was maintained. The Local Research Ethics Committee advised that formal ethics approval was not required as participants were not included as NHS users or employees.

### Evaluation aim and objectives

The aim of the study was to assess the feasibility, costs and effects of implementation of the HLA in two deprived communities in Pembrokeshire.

Objectives were to:

1. Support communities to work in partnership to identify needs, develop and disseminate Local Action Plans (LAPs) and to describe and assess these processes against the pre-agreed timetable

2. Assess individual and community impact from the HLA, including

• changes in health-affecting community-identified issues, which were perceived by residents, forums and stakeholders across key partner agencies to have occurred or been planned as a result of the intervention

• processes of liaison and influence between the forums and other agencies concerning implementation of action plans as perceived by forum members and stakeholders across partner agencies

3. Measure the use of resources from each partner organisation and individuals from the community in delivering the intervention

## Methods

Within the overall framework of the action research approach, a multi-method study design was utilised, to match the diverse study objectives. Main methods of data collection were:

• Documentary sources

○ minutes of meetings, reports, newsletters, correspondence

• Diaries and reflective accounts kept by Action Researcher

○ Records of project and community forum activity, including frequency, type, attendance, and content of individual and group contacts

• Face-to-face interviews

○ Community residents at outset of study (2001) and before end of study (2004) - random samples of residents and 'hard-to-reach' residents, as defined locally by the forums, were taken in each community. Sampled residents were contacted by post and then visited by a community researcher. Community researchers also interviewed each other since all were local residents. A semi-structured interview schedule was used which covered demographics; general health status, using SF36 [22,23]; views about healthy living and local issues of priority. The second interview also covered awareness of the project and perceptions of change since the first interview.

○ Key stakeholders - semi-structured interviews were carried out by appointment

• Focus groups using topic guides related to study's objectives, with

○ Community researchers

○ Community forum members - held as a discrete element of regular meetings

○ PSG - held as an extraordinary PSG meeting

• Costing proformas completed for

○ time related to project implementation for action researchers; statutory and voluntary staff ; and community researchers

○ travel

○ venues

○ other costs

### Setting

The Pembrokeshire SHARP project focused on two 'deprived' communities where existing local forums were already involved in healthy living activities.

At study outset, both areas exhibited high scores on the major deprivation indices including Oxford, Breadline Britain, Townsend, Jarman and Carstairs and with elevated Standard Mortality Rates, Standard Long Term Illness and permanent sickness ratios. [19,20] In each, a community group had been established to improve residents' health and wellbeing through targeted activities. Both communities had distinct historic identities but were now each subsumed as areas of a nearby larger town. Both areas were served by economic and community development projects including Communities First, Surestart and Employment Action Teams [24,25].

*Community 1*'s character changed when most of the small terraced cottages were cleared post-war for a new council housing estate. The second phase of the estate, still mostly in local authority ownership, had a high density of buildings separated by narrow alley-ways: houses and gardens were small and overlooked each other. There was a semi-permanent travellers' site nearby. Population was 1683 [26] and it was recorded as most "deprived" in Pembrokeshire [21]. The community forum was a residents' association, recently relaunched with local authority support, to foster community cohesion and generate environmental and social benefits.

*Community 2 *had a population of 4699 [26], living in council estates and older terraced streets. New executive-style homes, often with panoramic sea views, were enjoyed by locals who returned to the area in later life. It was the county's seventh most deprived area and third for educational deprivation [21]. The community forum was established with support from health promotion workers and aimed to improve community health through provision of information and group activities.

### Data collection and analysis

Documents, diaries and reflective accounts were gathered throughout the project and analysed narratively, in order to produce the 'story' of implementation. Interview schedules were developed in accordance with project objectives. All interviews were taped and transcribed with respondents' full consent. Coding was undertaken for structured data collection e.g. closed questions during interviews, SF36. Data were entered onto SPSS for descriptive analysis. Thematic analysis was used for all interview transcripts, an ongoing process based on collaborative discussion. Two or three members of the research team, including interview facilitators and observers, read transcribed interviews individually, made notes on key concepts and wrote a brief overview of essential categories and themes. Overall study themes from all qualitative data were debated and agreed by all study partners (community, statutory and voluntary) in a group discussion [27].

Resource use data were collected from designed proforma which were completed by project members throughout the study. Entries were recorded for project implementation activities only, with costs of overall project evaluation excluded. Resources were valued in money terms using the principles of economic evaluation [28] and expressed in 2004 prices. Costs were both for resources directly paid for e.g. the time of SHARP project staff, as well as those not directly paid for e.g. inputs by Local Authority staff, on the principle that these incur opportunity costs. Volunteers (community members and others who freely gave their time) are acknowledged as a vital resource in the intervention. Their time was recorded but not valued in money terms as part of the costing exercise.

## Results

### • Response rate, participant characteristics and missing data

Interviews by community researchers with residents during year 1 achieved a 44% response rate (n = 92). At year 4, attempts were made to re-contact those interviewed and 29 residents were re-interviewed in the two communities. A new sample of hard-to-reach residents from community 1 was sought because of the high turnover of people in this group (defined as council tenants in a discrete area); 14 were contacted and 10 interviewed. The total response rate at interview 2 was 42%. See table 1 for a full description of respondents.

**Table 1:** Who was interviewed in the communities? Response rates and sample characteristics

	Community 1 general population	Community 1 hard-to-reach	Community 2 general population	Community 2 hard-to-reach	Community researchers	Total combined sample
Total interviewed/contacted after exclusions*(%)						
Interview 1	33/70 (47%)	5/14 (36%)	35/69 (51%)	11/13(85%)	8/8 (100%)	92/174 (44%)

Interview 2	17/33(52%)	10/14 (71%)	11/69(16%)	1/11(9%)	Not repeated	39/84 (46%)

***Demographics: *Women (%)**						
Interview 1	25 (76%)	4 (80%)	16 (46%)	5 (50%)	8 (100%)	59 (64%)

Interview 2	14 (82%)	6 (60%)	7 (64%)	1 (100%)	Not repeated	28 (72%)

**Age range: number**						
Interview 1	16-25: 4	16-25: 2	16-25: 1	<16: 2	16-25: 1	16-25: 10
	26-45: 12	26-45: 3	26-45: 12	26-45: 2	26-45: 4	26-45: 33
	46-65: 15	46-65: 0	46-65: 7	46-65: 1	46-65: 3	46-65: 26
	66+: 2	66+: 0	66+: 11	66+: 2	66+: 0	66+: 15

Interview 2	Not known: 0	Not known: 1	Not known: 0	Not known:	Not repeated	Not known: 1
	16-25: 1	16-25: 1	16-25: 0	<16: 0		16-25: 2
	26-45: 6	26-45: 5	26-45: 1	26-45: 1		26-45: 13
	46-65: 9	46-65: 3	46-65: 9	46-65: 0		46-65: 21
	66+: 1	66+: 0	66+: 1	66+: 0		66+: 2

**Mean years lived in area**						
Interview 1	23.7	17.8	28.2	27.3	23.2	24

Interview 2	Not asked	Not asked	Not asked	Not asked	Not repeated	Not asked

**Employed/self employed (%)**						
Interview 1	13 (39%)	2 (40%)	15 (43%)	6 (55%)	7 (88%)	43 (47%)

Interview 2	8 (47%)	3 (30%)	5 (46%)	1 (100%)	Not repeated	17 (44%)

**Own home (%)**						
Interview 1	16 (49%)	0 (0%)	20 (57%)	10 (91%)	6 (75%)	52 (57%)

Interview 2	8 (47%)	0 (0%)	11 (100%)	1 (100%)	Not repeated	20 (51%)

**Some/fluent Welsh language (%)**						
Interview 1	6 (17%)	1 (20%)	2 (6%)	3 (27%)	2 (25%)	14 (15%)

Interview 2	6 (35%)	0 (0%)	3 (27%)	1 (100%)	Not repeated	10 (26%)

*Exclusions = dead, ill, illness in family, moving away, on holiday, vacant property, not known at address, known to be violent

At the end of the study, focus groups were carried out with:

○ community researchers (CRs): four (of original nine) who participated in administering the repeat interviews

○ each community forum (CF): three of a possible 11 attended from CF1 and seven of a possible 10 from CF2, reflecting usual attendance at forum meetings in each site

○ PSG members: six members attended, of a possible 20; one representative from each of the communities, one statutory body member, one voluntary sector representative and two academics from the external evaluation team

Interviews were carried out with external stakeholders to the project: six completed interviews of 18 contacted - two declined to participate; seven did not respond in time to participate; one withdrew without explanation. Responders represented key public sector organisations including the County Council and the Local Health Board (Welsh equivalent of Primary Care Trusts). The voluntary sector was represented by several interviewees. Everyone interviewed held a middle-to-senior management position.

The following codes are used to identify interview respondents. Respondents quoted are identified by a number following the relevant letter(s)

R: Resident

CR: Community Researcher

C: Community (numbered 1 or 2)

CF: Community Forum (numbered 1 or 2)

PSG: Project Steering Group

S: External Stakeholder

Community 1 Hard-to-reach = tenants on an 'undesirable' estate

Community 2 Hard-to-reach = housebound elderly people and shift workers

### Objective 1: To support communities to work in partnership to identify needs; develop and disseminate Local Action Plans (LAPs) and to describe and assess these processes against the pre-agreed timetable

**Figure 1:**
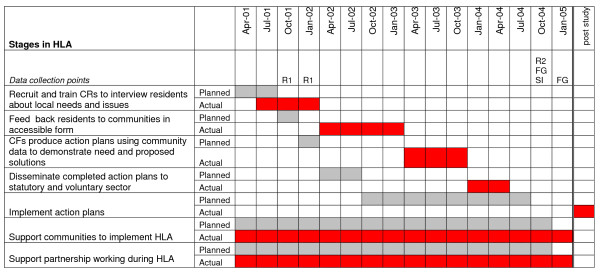
**time line of planned and actual activity**. *Data collection points *R1 First resident interviews R2 Repeat resident interviews FG PSG and CF focus groups for project evaluation SI Stakeholder interviews for project evaluation

In each community, local residents were recruited (C1 n = 4; C2 n = 5) and trained in interviewing skills. Interviews were carried out with residents. In each community, interview results were analysed by the community researchers and AR and presented in accessible formats to the community forum. Supported by the AR, each forum developed a LAP in response to interview results. From presentation to action plan launch took two years in each community, much longer than was anticipated at the project planning stage. Figure 1 shows the timeline of planned and actual activity stages in the HLA.

During years 2 and 3, monthly meeting frequency and attendance declined, according to meeting minutes and project records, as both forums lost membership including key members and committee officers so that meetings were not quorate with fewer than five participants. CF2 held "make-or-break" meetings four times in those two years. Each meeting generated short-term enthusiasm until participation declined, viability was questioned and the cycle repeated. Both CFs reported feeling let down by funding initiatives which were unrealised and the withdrawal of professional workers' support (Action Researcher notes). In written accounts of individual and group contacts, the Action Researcher recorded that some spoke of empty promises: *"it was only talk" *(AR notes), which was threatening community confidence:

*"we've been messing around for two years, this business of making improvements...that's why we've lost the goodwill of the people." *(AR notes)

With AR support, forums used action planning as an opportunity to engage residents and communicate investment needs to funding bodies. The Action Researcher noted that each forum held action planning workshops when local people watched a video made by community researchers in which interview results were presented by them using a journalistic 'in-field' style. One was a public event, attended by 40 people; the other a forum meeting attended by nine members. Issues raised were incorporated into local action points, then circulated and reconsidered at monthly or bimonthly meetings over six months where participants reviewed recommendations and wrote sections of the action plans (AR notes). The completed plans were printed and presented to up to 50 stakeholders through two local workshops, planned and delivered by community members. Forum members said the support was crucial in completing the action planning:

*"We wouldn't be here now if it wasn't for SHARP...that's what's brought the action plan" *(CF2 4)

### Objective 2: individual and community impact from the HLA - impact and processes of liaison

#### Views about healthy living

Healthy living was reported to be important to nearly all respondents in both communities and at each interview (see table 2). An overwhelming majority also reported that they understood how to live healthily, although the proportion reporting that healthy living was important to them or that they thought they lived in a healthy way was lower in community 1 (Fisher's exact test p = .003; .047). There were no differences in community 2 or between responses over time.

**Table 2:** Views on healthy living

	Agree/responded to the question	
***"Healthy Living is important to me"***	**Interview 1**	**Interview 2**

Community 1 general	33/37	16/17

Community 1 hard-to-reach	5/5	10/10 **

Community 2 combined sample*	46/48	11/12

***"I feel I understand how to live in a healthy way"***		

Community 1 general	30/34	17/17

Community 1 hard-to-reach	5/5	8/10 **

Community 2 combined sample*	46/49	12/12

***"I think that I live in a healthy way"***		

Community 1 general	25/34	12/17

Community 1 hard-to-reach	2/5	8/10 **

Community 2 combined sample*	43/48	11/11

* samples were combined to protect identity due to small numbers in samples at second interview

** at interview 2, a new sample of hard-to-reach residents was sought because of the high turnover of people in this group. There were fewer exclusions in the second sample, resulting in a larger final sample size than for interview 1.

#### General health status

Levels of self reported health status were similar or lower than might be expected in a general population sample [23] (see table 3).

**Table 3:** General health status - SF36 scores compared to published general population norms (at baseline - interview 1)

	n		Physical functioning	Social functioning	Role limitation physical	Role limitation Emotional	Mental health	Vitality	Pain	General health perception
*General population * [23]	*542*		*79.2*	*78.6*	*76.5*	*75.0*	*73.7*	*61.2*	*76.9*	*68.7*

SHARP community 1 (combined sample)	Max = 35		70.46	70.00	69.85	76.47	67.5	55.0	70.21	62.21

Difference (95% CI for difference)			8.74 (-4.29, 21.77)	8.60 (-3.75, 20.95)	6.65 (-8.33, 21.63)	-1.47 (-15.93, 12.98)	6.2 (-1.72, 14.12)	6.2 (2.90, 15.30)	6.69 (-5.45, 18.83)	6.49 (-3.22, 16.20)

SHARP community 2 (combined sample)	Max = 40		66.32	62.5	62.5	72.22	68.24	54.21	63.03	56.85

Difference (95% CI for difference)			12.88 (2.25, 23.51)*	16.1 (4.71, 27.49)*	14 (-1.25, 29.25)	2.78 (-12.16, 17.72)	5.46 (-2.43, 13.35)	6.99 (-0.04, 14.02)	13.87 (3.98, 23.76)*	11.85 (3.05, 20.65)*

*differences significant at 0.05 level by t-test

#### Problem issues

At first interview, respondents were asked to prioritise problem issues. Problems most frequently cited as highest priority were: crime (26 people), money (24 people), work (14 people), traffic (14 people), litter (11 people), local services and facilities (11 people), and housing (8 people).

Crime remained a concern for many at second interview, although perceptions of change varied. The majority of respondents reported no change or a worsening crime situation and, as in early interviews, listed petty crime, drunken street fighting, vandalism and antisocial behaviour - mostly linked with young people - as their highest priority problems. Residents reported that a new CCTV system had been installed locally to address fear of crime and they welcomed a temporary initiative to increase beat police but criticised it for being short term. Rumoured plans for a permanent local police presence were treated cautiously.

*"is it going to stop the crime?" *(R6)

Perceptions of change relating to money also varied. Many respondents linked money issues with work. Some reported changed personal circumstances, impacting on the importance of financial worries e.g. leaving work because of illness or no longer seeking work because of childcare commitments. Confidence about employment was low with respondents expressing concern about the lack of available work, much of which was seasonal or short-contract, and recent large-scale job losses.

*"A few people get some good work for a few months and then it's back to nothing again. When they talk about here as a deprived area and that there's funding for this and funding for that, it would be nice for something to actually exist." *(R24)

With regard to the problem of traffic, there were different views between communities. In community 1, more than half of respondents reported no change and maintained strong criticism of volume and speed of vehicles even though they knew that speed cameras had been installed to reduce pedestrian safety worries.

*"I don't think it's made much difference."*(R27)

However, respondents in community 2 identified improvements relating to traffic, some praising a new one-way arrangement on a large estate.

Changes related to the problem of litter were reported in both communities. A council clean-up of dumped household items around community 1 was praised but also criticised for being a one-off event. In community 2, almost all respondents described an improvement, listing a new kerb-side recycling scheme, regular street sweeper and more recycling bins locally, although dog fouling remained a problem.

Some improvements in local services and facilities were noted e.g. the opening of a new skateboard park, although others had closed e.g. children's football club. Desire for activities across the age range was expressed. Overall lack of facilities, particularly for young people, continued to be a concern.

*"Where are these kids going to go? They're going to be wandering the streets, they've got nothing to do." *(R36)

Housing, raised as a priority issue in community 1, was noted to have changed although views were mixed about how much had improved. Availability of housing was reportedly better but local authority properties remained an eyesore. Some external painting had brightened up the area and the litter and household items still dumped in gardens and lanes were now cleared away more quickly. New street lighting, fencing and a new recycling scheme were praised. But it was reported that the social problems linked to estate layout still existed and nothing had come of estate redesign proposals.

*"Nothing's happened. There's still loads of houses on this estate. They said they would knock them down, they haven't." *(R14)

#### Awareness of the SHARP HLA project

Two thirds of respondents said that they were aware of the community action plans and most (19/24) believed that they could potentially make a difference.

*"If they really believe in it and want to follow it through, then yes I really do believe that the voice of the people can make a difference." *(R34)

#### • Impact of HLA process on community HLA participants

Community researchers said the interview process had raised their awareness of the complexity of their communities. While they disliked the term 'deprived' to describe the place they said they called home, they also understood that hardship could make a community invisible and saw the interviews as giving a voice to people,

*"to have their views heard...because normally they are never listened to*" (CR2)

They reported having gained qualifications, skills and opportunities through involvement with the HLA project, which they said boosted their confidence and in some instances changed their outlook. One said the experience had led to her obtaining a new job; another had become actively involved in her community as an office-holder on two committees. Respondents reported that family members, friends and colleagues had seen a difference in them.

*"Without the project I don't think I would have done those things."*(CR1)

*"It has given us credibility." *(CR4)

Forum members felt their groups had been strengthened by the new experiences gained through the HLA.

*"We know what we are about now so we know how to tackle it...we've all highlighted that."*(CF2 6)

Data from interviews with residents had confirmed some issues but challenged other opinions, for example changing the approach of one forum member who was also a local councillor.

*"People have got different perceptions of what their priorities are...so it's changed my mind." *(CF2 2)

Forum members said they felt empowered and optimistic. They believed the action planning process had strengthened their case for changes on the ground and believed that the action plans were being heard by those in power. They listed specific changes resulting from the HLA as evidence of progress: the re-establishment of a youth club *"thanks to SHARP" *(CF1 3), extra street lighting; CCTV and proposals for improvement of a housing area with particular problems. Forum members were positive about the potential for further impact, with the action plan having been adopted by the government-initiated 'Communities First' programme in Community 1.

Individuals from the community forums who had found the language and format of joint meetings intimidating and unclear said they were slowly gaining the confidence to participate.

*"I was just a little bit put off. It was a bit heavy...They were talking a different language to me. You know, I just didn't get in there at all." *(CF2 4)

### • Impact on processes of liaison and influence

The HLA process was felt by community members to have brought local needs onto a wider agenda. Forum members believed the action plans had positively influenced their relations with statutory organisations and would continue to do so.

*"It has lit that fire which is causing some people to move a little bit uncomfortably." *(CF1 2)

*"They didn't know that different areas has (sic) got problems...it made people sit up and listen for once." *(CF2 6)

Project partners from the statutory and voluntary sectors reported confidence in the community research method used during the project for engaging residents, including hard-to-reach groups, and providing data which were seen to be credible. They reported raised awareness of community issues and perspectives amongst their colleagues. Engagement of external stakeholders was reported to have been poor initially because the nature and aims of the study were not well understood particularly within the local authority, but doubts had been overcome as confidence was gained in the process and quality of community involvement. Respondents from the statutory and voluntary sector said the community-led process had credibility which gave them confidence in the findings.

*"I think it's brilliant! It's actually the way to do it isn't it? It's all very well for health professionals to try and engage the public, but you're probably more likely to get real data, real information, if you're using the community to elicit the information." *(S4)

Representatives of commissioning agencies who sat on the Project Steering Group said the process had changed ways of thinking and working because it highlighted the community perspective.

*"You begin to turn the whole thing around. Very often we're guilty, thinking that it's our world and 'fit into our world' when of course it's really the other way around." *(PSG 3)

They reported individual examples of impact: incorporating the model as a trial in social care commissioning; a new partnership group to address community concerns; a successful £75 k joint statutory-community Healthy Living grant bid funded by the Welsh Assembly Government which was based on project findings.

However, ineffective internal communication was reported to have prevented consideration of project data in strategic planning and external stakeholders said managers questioned project relevance to health policy and short-term organisational priorities in the face of budgetary constraints and statutory responsibilities. It was acknowledged that no established mechanism existed in commissioning organisations for responding to the local voice.

*"The weakest aspect is the mechanism to feed into organisations - and whether that is a weakness of the project or a weakness of the organisations, I don't know." *(S4)

Neither did the infrastructure exist within statutory organisations to sustain the HLA process. External stakeholders therefore seemed to doubt that the process would have any lasting impact. Community members were also cautious about sustainability, reporting that optimism was tempered by unsuccessful experiences and loss of the Action Researcher's support.

*"There's been X amount of money gone into it, a lot of work, a lot of blood, sweat and tears, and expectations raised... but who picks up the next thread?" *(S3)

### Objective 3: To measure the use of resources from each partner organisation and individuals from the community in delivering the intervention

Resource use data for implementing the SHARP project were gathered from November 2001 through December 2004. The first 59 weeks were costed separately as they can be regarded as 'set-up' costs. All costs are expressed in 2004 prices.

For the two communities, set-up costs came to £50,730 (table 4) and costs of the following two years to £65,878 (table 5). Total cost of the project from the start to the end of the reporting period was thus £116,608 or an average of £58,304 per community.

**Table 4:** Set-up costs: 59 week period

	SHARP staff hours (cost)	Community researchers hours (cost)*	Other volunteer hours	Professional hours (cost)	Travel miles (cost)	Materials venue & other (cost)	Total cost
**Project Meetings**	125 (£2017)	0	39.5	67.5 (£1526)	1410 (£622)	£92	£4257

**Community Meetings**	170 (£2749)	0	80	83 (£1453)	882.5 (£390)	0	£4590

**Meetings Statutory Bodies**	42 (£669)	0	0	267 (£3821)	107 (£47)	0	£4538

**Recruiting Researchers**	125.5 (£2027)	0	90	16 (£333)	338 (£149)	£591	£3097

**Training & Managing Researchers**	187 (£3019)	307	89	14 (£420)	910 (£387)	£4560	£8386

**Interview Preparation & Conducting**	121 (£1954)	224 (£4121)	143.5	4 (£65)	2221 (£980)	£204	£7324

**Analysing Data**	269 (£4341)	249	0	105 (£1828)	578 (£255)	£67	£6491

**General Duties**	745.5 (£12,046)	0	0	0	0	0	£12,047

Total **	1785 (£28,813)	780 (£4121)	442	556 (£9,446)	6447 (£2,830)	£5515	£50,730

* Hours were not costed as community researchers gave their time voluntarily. They were, however, paid for each completed interview. Amount shown represents the money paid to them.

** Inexact sums due to rounding

**Table 5:** Costs for 2 year period following set-up.

	Community Researcher voluntary hours	Statutory worker hours (cost)	SHARP staff hours (cost)	SHARP staff travel minutes (cost)	SHARP staff travel miles (cost)	Other costs **	Total cost
**Action planning**	140	71 (£1289)	545 (£7673)	1555 (£450)	721 (£296)		£9,708
**Dissemination**	458	152 (£2759)	599 (£10,584)	2416 (£731)	737 (£302)	£1801	£16,177
**Unallocated**	226	44 (£799)	2204 (£32,002)	12,752 (£3479)	3556 (£1458)	£2255	£39,993
**Total**	824	267 (£4847)	3348 (50,259)	16,723 = 279 hours (£4660)	5014 (£2056)	£4056	£65,878

** Other costs included marketing, translation, printing and refreshments

## Discussion

### • Summary of key findings

The HLA project achieved its objective of supporting communities to work in partnership in the process of identifying their own needs, producing and disseminating action plans, although the process took longer than anticipated.

The process allowed residents to define the issues considered to impact on health and wellbeing, with a list ranging from social activities and youth facilities to employment issues, quality of the community environment, traffic and crime. Commissioning organisations reported confidence in the message from communities.

Feelings about individual and community impacts were mixed. Due to project implementation having been slower than expected, evidence of impact related to the action plans was not sought. Community residents were aware of the community action plans and most agreed cautiously that they could make a difference. Community members personally involved in the project gained skills, insight, confidence and a greater sense of community spirit. Community members and organisations felt the process gave communities a more effective voice which statutory and voluntary sector agencies were listening to and some impacts were described. Commissioning managers had made some steps towards responding to the action plans - plans and funding bids, for example - suggesting the project influenced local policy decisions and delivery of services in a way which could create some form of legacy.

Conclusions regarding cost effectiveness are not possible given the project's multiple objectives and it was always beyond the scope of the evaluation to attempt to draw conclusions regarding whether the project represents an efficient use of resources (value of benefit exceeds value of costs). However, it is important that the achievements of the Pembrokeshire SHARP project can now be discussed in relation to what it costs. At an average cost per community of £25,365 to set up and £32,939 per year thereafter the project was not particularly costly, especially if seen in the context of other expenditures, for example a total budget for Pembrokeshire County Council of over £300 million per year.

### • Implications of study

Taking a whole-community approach to healthy living, drawing on existing resources rather than focusing on an actual building, was a novel element of this project [29]. The study has shown that the intervention is feasible, although not within the original timescale set for its implementation. The difference between anticipated and actual pace challenged expectations and working practices for the funders, local agencies and the participating communities. This has a clear message for policy and practice - engagement of communities in a process such as this one can be done but it takes time and cannot be rushed, as suggested by previous authors from UK and international studies [30-32]. The process demonstrated that capacity exists for volunteers to take on research, planning and partnership working roles [33]. However, community ownership requires working at the pace of the community members, who are generally volunteering their time and cannot work to the timetables of health, social care or community development professionals [4].

The HLA appears to have given meaningful opportunities for community members to engage in community-based health initiatives. Confidence in the process among all partners was slowly built but key to multi-sector partnership working. The participative method, focused on generating action through a multi-sector partnership, seems a viable and appropriate one for tackling the compounding effects of local agencies and raising awareness of the community voice and community capacity [34,35]. It also allowed community members to define the inter-related factors that affect health and the mix of community, environmental and economic factors influencing individuals' perceived health and wellbeing.

As far as has been possible, reported costs reflect what the cost to other communities would be if replicating SHARP. Thus costs related to the evaluation aspects of action research which are an integral part of the intervention have been included but those associated with the evaluation of the project as a whole have been excluded. The geography of Pembrokeshire, the location of the two communities and the experimental nature of the project suggest that the travel costs incurred in SHARP - which includes the value of the time spent travelling (£9,546 or 8% of total project costs) may overstate the cost of replicating the project in other communities.

The intervention successfully provided access to the community, gaining information valued for its authenticity and engaging community residents in joint working with agencies. Impact in terms of influence and liaison were mixed and sometimes unexpected. The longer timescale needed may be one reason for this [36]. It is also difficult to attribute effects [15]. Although specific examples of change were numerous, there was still an overall feeling of nervousness that all that effort might not have any lasting effects. Structures and pathways in statutory partners clearly did not support the process of communication and utilisation of results in organisational planning processes [37]. That there were in fact some organisational impacts suggests that change relies on individual support, which is vulnerable to political, economic and personal factors. Community confidence remains fragile, more influenced by collective memory and low expectations than briefly experienced changes [38,39].

Community members demonstrated they have the skills and confidence to undertake research and engage with the issues raised. There were clear indications of benefit to those involved in the process, although wider benefits were difficult to demonstrate at this stage. Longer term assessment of outcomes is necessary [14,40,41].

### • Study limitations

The before and after study design means that the validity of findings could be threatened by concurrent events causing change and uncertainty about attribution, as highlighted in the introduction [15]. However, the benefits of the method used were judged by study collaborators across agencies and levels to outweigh these challenges [14,42]. In practical terms, neither an RCT nor a controlled before and after design could be implemented, due to the nature of the intervention and its context. The pragmatic nature of the adopted study design was as high as was possible to achieve on the hierarchy of evidence scale and its limitations have been taken into account in the interpretation and reporting of results.

We randomly sampled community residents and purposively sampled hard-to-reach groups, to reduce risk of selection bias. In addition, community researchers followed protocols for contacting the sample. The action research approach taken in this study was felt to have increased the participation rates across the project, but overall response rates were still low, introducing the risk of selection and attrition bias, limiting their usefulness. This may have been due to competing pressures and priorities for the action researchers to achieve a successful implementation of the HLA intervention; due to the inexperience of community researchers; or due to the challenging context for the study. In particular, the low response rate at second interview was largely due to lack of availability of the community researchers. Given the delays in implementation and overall timescale, the dynamic and mobile nature of the workforce and the community up-skilling during the study, several individuals had moved on to new roles.

We used semi-structured interview schedules to reduce risk of interviewer bias across the team. Throughout data collection and analysis, we made sure that we assessed our findings according to the study contexts and that issues studied could be clearly linked to the participant group. We made sure to question any ambiguous responses and to consider whether the responses gathered were relevant to the whole group and/or the individual providing the answers. Data were assessed by the study team and Project Steering Group who were asked to raise any issues of concern. No specific issues arose regarding any potential sources of bias, including recall bias and individual source bias [43]. We also included verbatim quotations in our findings to illustrate how respondents described events [44].

The total cost figures represent economic rather than financial costs i.e. they reflect the value of the resources used and include resources drawn on by the project but funded from other sources (statutory worker time). Although clearly an essential part of the HLA, the 2046 voluntary hours (including set-up) given to the project by the community researchers have not been valued in money terms and are not included in the cost figures above. There are various ways that volunteer hours could be valued [28]. Using a shadow wage of £4.85 per hour (2004 UK minimum wage for adults) the value of the volunteer hours is £9,923. This - after subtracting the £4121 paid to the community volunteers to avoid double counting - has the effect of increasing the total cost of the project to £122,410 or £61,205 per community.

Due to delays in implementation of the HLA intervention, the planned assessment of outcomes in the communities was not appropriate. Data were still gathered in each community concerning local priorities and awareness of the SHARP project, but a longer term follow up of outcomes would still be required to fully assess community gain.

## Conclusions

Overall, the SHARP HLA intervention was shown to be feasible and relatively inexpensive, with some encouraging indications of early impact at individual, community and local policy planning levels. However, clear messages from this study are that this is a long term process, with long term investment and evaluation required. To be fully effective, the intervention needed to have been embedded in cross- and within-partner planning and service delivery processes. These findings echo and build upon previous work in this area and have implications internationally for policy makers and practitioners who are working to tackle health inequalities using a 'grass roots', service user-focused approach.

## Competing interests

The authors declare that they have no competing interests.

## Authors' contributions

HAS led design of the study, undertook quantitative analysis, contributed to focus group analysis and led the drafting of the manuscript. BAE was the Action Researcher and supported implementation of the intervention, contributed to study design, coordinated all data collection, undertook focus group data collection and analysis and contributed to drafting the manuscript. DC led the design and analysis of the economic aspects of the study and contributed to drafting the manuscript. MN contributed to study design, undertook and analysed the stakeholder interviews and contributed to drafting the manuscript. FR provided advice throughout the study related to qualitative research methods; undertook and analysed interviews and contributed to drafting the manuscript. JS led partnership involvement in the study, contributed to study design, advised on implementation and contributed to drafting the manuscript. AM and TD were community researchers and forum members and undertook data collection, contributed to analysis, project management and drafting the manuscript. D O'S undertook study administration, contributed to analysis and drafting the manuscript. All authors read and approved the final manuscript.

## Pre-publication history

The pre-publication history for this paper can be accessed here:

http://www.biomedcentral.com/1471-2458/11/25/prepub

## References

[B1] Department for Communities and Local Government Stronger and prosperous communities - the local government white paper 2006 London: Department for Communities and Local Government

[B2] Department of Health Choosing health: making healthy choices easier 2004 London: Department of Health

[B3] Electoral Commission Social exclusion and political engagement: research report 2005 London: Electoral Commission

[B4] NICE Community engagement to improve health: public health guidance 9 2008 National Institute for Health and Clinical Excellence: London

[B5] Barnes M Health Action Zones: Partnerships for Health Equity 2005 London: Routledge

[B6] Brooks F Munro J Thomas K Heart Of Our City final evaluation report 1996 Sheffield Hallam University

[B7] Welsh Assembly Government Interim evaluation of Communities First: Final Report 2006 Welsh Assembly Government: Cardiff

[B8] Oakley P Pratt B Clayton A Outcomes and Impact: Evaluating Change in Social Development 1998 Oxford: Intrac

[B9] Minkler M Wallerstein N Minkler M and Wallerstein N Introduction to Community Based Participatory Research Community-Based Participatory Research for Health 2003 Jossey-Bass: San Francisco

[B10] Cheatham A Shen E Minkler M and Wallerstein N Community Based Participatory Research with Cambodian Girls in Long Beach, California: A Case Study Community-Based Participatory Research for Health 2003 Jossey-Bass: San Francisco

[B11] Welsh Office Better Health Better Wales 1998 Cardiff: Welsh Office

[B12] NICE Community engagement and development approaches to health: consultation on the synopsis of the evidence NICE 2007

[B13] Petticrew M Roberts H Evidence, hierarchies and typologies: horses for courses Journal of Epidemiology and Community Health 2002 57 527 529 10.1136/jech.57.7.527 PMC1732497 12821702

[B14] World Health Organisation The Ottawa Charter: principles for health promotion 1986 Copenhagen: WHO Regional Office for Europe

[B15] Hills D Evaluation of community level interventions for health improvement: a review of experience in the UK 2004 Health Development Agency: London

[B16] Wallerstein N Duran B Minkler M and Wallerstein N The Conceptual, Historical and Practice Roots of Communtiy Based Participatory Research and Related Participatory Traditions Community-Based Participatory Research for Health 2003 Jossey-Bass: San Francisco

[B17] Reason P Bradbury H eds The Sage Handbook of Action Research: Participative Inquiry and Practice 2008 2 Sage Publications Ltd: London

[B18] Quinn Patton M Qualitative Research and Evaluation Methods Michael Quinnn Patton 2002 2002 3 Thousand Oaks, California: Sage Publications

[B19] Dyfed Powys Health Authority Inequalities in Health 2000 Dyfed Powys Health Authority

[B20] Pembrokeshire County Council Indicators of Poverty and Deprivation 1996 Pembrokeshire County Council

[B21] Welsh Assembly Government Welsh Index of Multiple Deprivation 2000 Welsh Assembly Government

[B22] Brazier JE Validating the SF36 health survey questionnaire: new outcome measure for primary care British Medical Journal 1992 305 160 164 10.1136/bmj.305.6846.160 1285753 PMC1883187

[B23] Garratt AM The SF-36 health survey questionnaire: an outcome measure suitable for routine use within the NHS? British Medical Journal 1993 306 1440 4 10.1136/bmj.306.6890.1440 8518640 PMC1677883

[B24] Department for Children Schools and Families http://www.dcsf.gov.uk/everychildmatters/about/surestart/surestart/

[B25] National Assembly for Wales Skills and Employment Action Plan for Wales 2005 2005 Welsh Assembly Government: Cardiff

[B26] Office for National Statistics Census of Population 2001 ONS: London

[B27] Van Manen M Researching Lived Experience 1990 Ontario. Canada: State University of New York Press

[B28] Drummond M Methods for the Economic Evaluation of Programmes in Health Care 2005 3 Oxford: Oxford University Press

[B29] Ashton J The Peckam Pioneer Health Centre: a reappraisal 1977 8 Community Health 132 138 322939

[B30] Dockery G de Koning K and Martin M Rhetoric or reality? Participatory research in the National Health Service, UK Participatory Research in Health 1996 Zed Books Ltd: London

[B31] Hanson E Nolan M, et al ACTION (Assisting Carers Using Telematic Interventions to meet Older people's Needs): practitioners' reflections on a Swedish innovation User Participation in Health and Social Care Research 2007 Open University Press: Maidenhead

[B32] Cropper S Goodwin M Cropper S, et al 'Policy experiments': policy making, implementation and learning Community health and wellbeing: action research on health inequalities 2007 The Policy Press: Bristol

[B33] Westhues A Developing theory from complexity: reflections on a collaborative mixed method participative action research study Qualitative Health Research 2008 18 5 701 717 10.1177/1049732308316531 18420539

[B34] Cropper S Cropper S, et al Action research partnerships: contributing to evidence and intelligent change Community health and wellbeing. Action research on inequalities 2007 Policy Press: Bristol 73 104

[B35] Bjorndal A Improving social policy and practice: knowledge matters The Lancet 2009 373 1829 1831 10.1016/S0140-6736(09)60783-2 19446328

[B36] Cameron A Lart R Factors promoting and obstacles hindering joint working: a systematic review of the research evidence Journal of Integrated Care 2003 11 2

[B37] Pickin C Developing a model to enhance the capacity of statutory organisations to engage with lay communities Journal of Health Service Research and Policy 2002 7 1 34 42 10.1258/1355819021927656 11822259

[B38] Oliver S Involving consumers in research and development agenda setting for the NHS: developing an evidence-based approach Health Technology Assessment 2004 8 15 1 148 15080866 10.3310/hta8150

[B39] Gowman N Healthy Neighbourhoods 1999 Kings Fund: London

[B40] Staley K Exploring Impact: Public involvement in NHS, public health and social care research 2009 INVOLVE: Eastleigh

[B41] Viswanathan M Community-Based Participatory Research: Assessing the Evidence Evidence Report/Technology Assessment No 99. AHRQ Publication 04-E022-2 2004 Agency for Healthcare Research and Quality: Rockville, MD PMC4780908 15460504

[B42] Nolan M Nolan M, et al Introduction: what counts as knowledge, whose knowledge counts? Towards authentic participatory enquiry User Participation in Health and Social Care Research 2007 Open University Press: Maidenhead

[B43] Robson C Real World Research 2002 Oxford: Blackwell Publishers

[B44] Coffey A Atkinson P Making Sense of Qualitative Data 1996 London: Sage Publications

